# Visual Acuity Changes during Pregnancy and Postpartum: A Cross-Sectional Study in Iran

**DOI:** 10.1155/2014/675792

**Published:** 2014-09-28

**Authors:** Khashayar Mehdizadehkashi, Shahla Chaichian, Abolfazl Mehdizadehkashi, Ebrahim Jafarzadepour, Zeinab Tamannaie, Bahram Moazzami, Mohaddeseh Pishgahroudsari

**Affiliations:** ^1^Pars Advanced & Minimally Invasive Manners Research Center, Pars Hospital, Tehran 1693736513, Iran; ^2^Minimally Invasive Techniques Research Center of Tehran Medical Sciences Branch, Islamic Azad University, Tehran 1693736513, Iran; ^3^Endometriosis Research Center, Iran University of Medical Sciences, Tehran 1693736513, Iran; ^4^Department of Optometry, Iran University of Medical Sciences, Tehran 1693736513, Iran; ^5^Shahid Beheshti University of Medical Sciences and Minimally Invasive Surgery Research Center, Iran University of Medical Sciences, Tehran 1693736513, Iran; ^6^Minimally Invasive Surgery Research Center, Iran University of Medical Sciences, Tehran 1693736513, Iran

## Abstract

In this research, we represent the changes in visual acuity during pregnancy and after delivery. Changes as myopic shift start during second trimester and will be stopped after delivery; however it is obtained that women will have the same refractive error as what they had in the first trimester, after postpartum. So, any change in their spectacle prescription during this period is forbidden. As a result, not only changing in hormones can cause myopic shift in vision, but also overweight has its retributive role. What we are trying to do is to notify gynecologists and optometrists to be aware of these changes, so as to leave spectacle prescription writing to the session after postpartum period.

## 1. Introduction

Visual acuity disturbances are common complaints in pregnancy suggesting the presence of underlying diseases including diabetes, preeclampsia, or refractive eye disorders [1–3]. However, most cases are just related to physiology of pregnancy and hence do not need serious medical treatment [4]. Physicians should be able to distinguish between the pathologic and normal physiologic changes of visions during pregnancy to avoid unnecessary medication or distressing diagnostic management [5].

Most studies have compared the effects of pregnancy on the outcomes of refractive surgeries [1–5]; however, to the best of our knowledge no single study has addressed the changing pattern of visual acuity in pregnancy and there is no evidence regarding the severity of visual acuity disturbances neither. Our study hence aimed to investigate visual acuity changes in pregnancy and to determine how the vision changes would be following the child birth. This study indeed will answer the long standing question about the pregnant women's visional disturbances.

## 2. Methods

A cross-sectional study was conducted in a university clinic of perinatology affiliated to Tehran University of Medical Sciences in Tehran, where pregnant women attended for screening and routine visits between 2010 and 2012. Pregnant women between 20 and 39 years of age were eligible to enter the study if there was no bilateral blindness, absence of accompanied medical conditions, and no history of medications used during pregnancy but the administered supplements. Those with abortion or who were lost to followup were excluded from the study.

Research ethics committee of TUMS reviewed and then approved the study protocol and informed consent was obtained from each patient before entering the study. Patients were examined for visual acuity in each trimester and 3 months after delivery by an optometrist using the Snellen chart in a 6-meter distance assessing the distance and a 40-centimeter chart for near vision. At each visit, patients' physical and clinical findings were also recorded in a designed questionnaire. Data were then analyzed by Statistical Package for windows (SPSS, 15, Inc., Chicago) using repeated measure ANOVA; the values were considered significant at *P* < 0.05.

## 3. Results

A total of one hundred and seventeen participants were enrolled in this study of which 5 pregnancies ended in abortion and 5 participants were lost to followup; hence, the remaining 107 pregnant women were analyzed in terms of visual acuity changes during and after pregnancy period. Mean ± SD age of patients was 31.49 ± 3.64 years. Eighty-three participants (77.5%) had college education. In addition, 98 participants (91.6%) lived in Tehran while the remainder nine ones (8.4%) were residents of other cities, so most of the participants were not in any bad condition such as fatigue feelings because of the road traffic, so it can show that our results are reliable. Participants' demographics are summarized in Table 1.

The participants' symptoms was assessed during pregnancy in 4 intervals in a perinatal clinic. The most common symptom during the first trimester was nausea occurred in 51 women (48.6%). And headache was the most common symptom during the second trimester which was occurred in 73 cases (76.1%). On the other hand, on second trimester, visual blurredness was most frequently seen in 91 participants (89.2%) of which most cases had occurred in the afternoon. Hence, by obstetrical examinations, no preeclamptic changes have been found in these cases. In the third trimester abdominal pain was the most popular complaint in this group of pregnant women (48 cases or 48%). In addition, flushing occurred most frequently in 54 participants (52.9%) in the third trimester.

Distance visual acuity in the right eye was significantly different between the three trimesters (*P* < 0.001) but it did not statistically differ between the early pregnancy (early first trimester) and postpartum period (*P* = 0.42).

Near visual acuity in the right eye differed significantly between the first and the second trimesters of pregnancy (*P* < 0.05) while its difference from the second trimester to the late pregnancy and later to the postpartum period was not statistically significant (*P* > 0.05) suggesting a decreased near acuity from the first trimester to the second trimester which then remained unchanged after to the late pregnancy and revealed postpartum.

Similarly, distance visual acuity in the left eye differed significantly from the first trimester to the second and from the second trimester to the third one (*P* < 0.001) while the acuity was not significantly different between early pregnancy and postpartum period (*P* > 0.05).

Regarding near visual acuity in the left eye, the changes between trimesters were statistically significant (*P* < 0.001); however, comparing the acuity between the beginning of pregnancy and postpartum period, no statistically significant changes were seen (*P* > 0.05).

Both binocular distance and near acuities showed statistically significant changes (*P* < 0.05) during pregnancy from the first to the third trimesters while the acuity returned roughly to its primary level after delivery with no significant difference between the early pregnancy and postpartum period (*P* > 0.05).

Respectively, at the second trimester and third trimester and postpartum, decrease in distance visual acuity in the right eye in 54 (54%), 72 (72%), and 8 (8.2%) of participants, decrease in near visual acuity in the right eye in 1 (1%), 10 (9.9%), and 4 (4%) of participants, decrease in distance visual acuity in the left eye in 59 (59%), 80 (80.8%), and 8 (8.2%) of participants, decrease in near visual acuity in the left eye in 12 (12%), 22 (22.2%), and 3 (3.1%) of participants, decrease in binocular distance visual acuity in 51 (51%), 74 (74.7%), and 8 (8.2%) of participants, and finally decrease in binocular near visual acuity in 11 (11%), 20 (20.2%), and 4 (4.1%) of participants were observed (Table 2).

Our study data suggests the visual acuity alterations during pregnancy, specially, starting in first trimester, can be caused by changes in the thickness of cornea which is believed to be due to hormonal changes during this period [1, 2]. The alterations were irrelevant to any preexisting visual disorders or low visual acuity as all participants were tested with their best corrections (wearing spectacles or contact lenses) during the study. The participants resumed their normal visual acuity after delivery.

## 4. Discussion

Impaired vision is of common complaints during pregnancy either alone or in association with other complications. As demonstrated in our study, headache and morning sickness are commonly associated with blurred visions. Our results are in the same line with the study by Pizzarello [6]: when visual examination of 95 pregnant women showed myopic shifts during pregnancy after delivery in both myopic and hyperopic changes, the visual acuityreturns to the amount close to prepregnancy. However, in our study, both myopic and hyperopic changes were observed with majority of the cases experiencing decreased acuity in either the distance or near visions. Our study in comparison with the other ones included larger population of pregnant women, followed them in closer intervals, and then compared the postpartum changes with primary visions.

There is paucity of knowledge regarding refractive errors related with pregnancy. On one hand, most reviews in ophthalmology discussed variety of the eye diseases in pregnancy with little attention to refractive disorders of the eyes [1, 2, 4–7]. On the other hand, obstetricians rarely if ever have discussed the pattern of visual acuity during pregnancy [8–10]. However, despite lack of sufficient evidences on the severity and reversibility of visual acuity and associated disorders under the title of eye refractive errors of pregnancy, authors have proposed serious concerns about visual disturbances during pregnancy [4–6]. Our study in contrast to these concerns showed that most of the refractive errors in near and distance vision of both eyes, if any, would be temporarily associated with gestational periods resolving significantly by child delivery. This could be of more importance when such refractive errors even exist prior to pregnancy leading to avoidance of unnecessary diagnostic processes.

Sunness reviewed health status of women's eyes during pregnancy with special attention to the preexisting conditions [2]. However, systematic refractive changes have never been mentioned in his review. Furthermore, the review by Weinerb et al. did not discuss the state of refractive errors in pregnancy neither [9]. Similar dearth is also observed in other studies [1, 3–5, 7, 8]. There is another report introducing transient myopia in a pregnant woman which resolved spontaneously 4 weeks after its onset [10].

Up to now, only Pizzarello has discussed refractive changes in pregnancy [6] and our study sits at the next systematically address this common but neglected phenomenon in women's eyes during a sensitive period. Although cataract, diabetes mellitus, and accommodative spasm have been mentioned as an explanation for visual acuity and refractive changes during pregnancy, more studies are necessitated to investigate the casualty of this incident. Our study with its larger sample size compared to the others can be an initiative of pregnancy complaints' clarification. However, more analytical design as a prospective cohort with respect to the documented etiologies could add to the validity and predictability of the future studies.

## Figures and Tables

**Table 1:** Demographic information of participants.

	Pregnant women
Education	
Under diploma	1 (0.9%)
High school diploma	23 (21.5%)
B.S.	65 (60.7%)
M.S.	13 (12.1%)
Ph.D.	5 (4.7%)
Home town	
Tehran	98 (91.6%)
Other cities	9 (8.4%)

**Table 2:** Visual acuity changes in 2nd, 3rd, and postnatal trimesters compared to 1st trimester.

	Pregnancy trimesters	No changes in visual acuity	Decreased visual acuity	Increased visual acuity
Distance visual acuity in right eye	Second trimester	46∗ (46%)	54 (54%)	None∗∗
	Third trimester	26 (26%)	72 (72%)	2 (2%)
	Postnatal	86 (88.7%)	8 (8.2%)	3 (3.1%)

Near visual acuity in right eye	Second trimester	101 (99.0%)	1 (1.0%)	None
	Third trimester	90 (89.1%)	10 (9.9%)	1 (1.0%)
	Postnatal	86 (86.0%)	4 (4.0%)	10 (10.0%)

Distance visual acuity in left eye	Second trimester	41 (41.7%)	59 (59.0%)	None
	Third trimester	19 (19.2%)	80 (80.8%)	None
	Postnatal	80 (81.6%)	8 (8.2%)	10 (10.2%)

Near visual acuity in left eye	Second trimester	88 (88.0%)	12 (12.0%)	None
	Third trimester	77 (77.8%)	22 (22.2%)	None
	Postnatal	92 (93.9%)	3 (3.1%)	3 (3.1%)

Distance visual acuity binocularly	Second trimester	49 (49.0%)	51 (51.0%)	None
	Third trimester	25 (25.3%)	74 (74.7%)	None
	Postnatal	81 (82.7%)	8 (8.2%)	9 (9.2%)

Near visual acuity binocularly	Second trimester	89 (89.0%)	11 (11.0%)	None
	Third trimester	79 (79.8%)	20 (20.2%)	None
	Postnatal	91 (92.9%)	4 (4.1%)	3 (3.1%)

*Number of patients.

∗∗“None” means visual acuity has reversed to its level on 1st trimester.
