# Corrigendum to “Visual Acuity Changes during Pregnancy and Postpartum: A Cross-Sectional Study in Iran”

**DOI:** 10.1155/2018/5280697

**Published:** 2018-02-20

**Authors:** Khashayar Mehdizadehkashi, Shahla Chaichian, Abolfazl Mehdizadehkashi, Ebrahim Jafarzadepour, Zeinab Tamannaie, Bahram Moazzami, Mohaddeseh Pishgahroudsari

**Affiliations:** ^1^Pars Advanced and Minimally Invasive Medical Manners Research Center, Pars Hospital, Iran University of Medical Sciences, Tehran, Iran; ^2^Minimally Invasive Techniques Research Center of Tehran Medical Sciences Branch, Islamic Azad University, Tehran 1693736513, Iran; ^3^Endometriosis Research Center, Iran University of Medical Sciences, Tehran 1693736513, Iran; ^4^Department of Optometry, Iran University of Medical Sciences, Tehran 1693736513, Iran; ^5^Shahid Beheshti University of Medical Sciences and Minimally Invasive Surgery Research Center, Iran University of Medical Sciences, Tehran 1693736513, Iran; ^6^Minimally Invasive Surgery Research Center, Iran University of Medical Sciences, Tehran 1693736513, Iran

In the article titled “Visual Acuity Changes during Pregnancy and Postpartum: A Cross-Sectional Study in Iran” [1], there was a mistake in the first affiliation. The corrected affiliations are shown above.
